# Interface engineering for facile switching of bulk-strong polarization in Si-compatible vertical superlattices

**DOI:** 10.1038/s41598-024-56997-0

**Published:** 2024-03-21

**Authors:** Pawan Kumar, Jun Hee Lee

**Affiliations:** 1 Department of Energy Engineering, School of Energy and Chemical Engineering, Ulsan National Institute of Science and Technology (UNIST), Ulsan, 44919 Republic of Korea; 2 Graduate School of Semiconductor Materials and Devices Engineering, Ulsan National Institute of Science and Technology (UNIST), Ulsan, 44919 Republic of Korea

**Keywords:** Energy science and technology, Materials science, Physics

## Abstract

Ferroelectric thin films incorporating different compositional layers have emerged as a promising approach for enhancing properties and performance of electronic devices. In recent years, superlattices utilizing various interactions between their constituent layers have been used to reveal unusual properties, such as improper ferroelectricity, charged domain walls, and negative capacitance in conventional ferroelectrics. Herein, we report a symmetry scheme based on the interface engineering in which the inherent cell-doubling symmetry allowed atomic distortions (phonons) in any vertically aligned superlattice activate novel interface couplings among atomic distortions of different symmetries and fundamentally improve the ferroelectric properties. In a materialized case, the ionic size difference between Hf^4+^ and Ce^4+^ in the HfO_2_/CeO_2_ (HCO) ferroelectric/paraelectric superlattice leads to these couplings. These couplings mitigate the phase boundary between polar and non-polar phases, and facilitate polarization switching with a remarkably low coercive field (${E}_{c}$) while preserving the original magnitude of the bulk HfO_2_ polarization and its scale-free ferroelectric characteristics. We show that the cell-doubled distortions present in any vertical superlattice have unique implications for designing low-voltage ferroelectric switching while retaining bulk-strong charge storing capacities in Si-compatible memory candidates.

## Introduction

Superlattices, which built as the layer-by-layer deposition of different functional materials, in ferroelectric thin films have potential applications in various fields^1^, including nanoelectronics^2^, data storage, energy storage^3^, and energy harvesting^4^, owing to their exceptional functional properties. These superlattices can help tune the ferroelectric properties, such as the polarization^5–8^, coercive field^9^, and stabilization of different domain walls^10^, thereby significantly enhancing device performance in sensors, transistors, and capacitors^11^. In recent years, ferroelectric-paraelectric superlattices^12^ in conventional ferroelectrics have been extensively investigated to tune the critical temperature (${T}_{c}$) of the transition between the paraelectric and ferroelectric phases, stabilize^13^ the charged domain walls^14,15^, realize negative capacitance^16,17^ and improper ferroelectricity^18^. However, the remnant polarization in such short period superlattices is significantly^19^ reduced, which limits their potential applications. To address this issue, we designed a ferroelectric-paraelectric vertical superlattice, which has not attracted sufficient attention despite its significant implications through symmetry engineering. This superlattice exhibits non-diminished polarization and can be experimentally grown, as demonstrated in vertically aligned nanocomposites (VANs)^20,21^.

In the recent discovery of scale-free ferroelectricity in HfO_2_^22^, which shows an unprecedented robust ferroelectricity down to single unit cell thin film^23^, it has been found that the domain wall energy is almost zero, and the ferroelectric polarization at the domain walls does not suppress. This unusual ferroelectric feature makes HfO_2_ a suitable candidate for non-volatile dense memory devices. The scale-free ferroelectricity in HfO_2_ is governed by flat polar phonon bands in its ferroelectric phase. However, polarization switching in HfO_2_ must cross an intrinsically very high energy barrier (1.34 eV) of ferroelectric domain wall motion^22^, the estimated energy barrier for uniform polarization switching via the tetragonal intermediate phase was 0.33 eV/u.c^24^ (u.c.: unit cell with four hafnium and eight oxygen atoms). These significantly large energy barriers limit facile switching in HfO_2_ for its applications in memory devices. Previous research^24,25^ have suggested that the reversal of polarization between the up- and down-polarized states of HfO_2_ generally passes through its cubic or tetragonal phases. However, the energies of these two phases are considerably larger than that of the polar orthorhombic phase. Thus, it is unlikely that the energy barriers for polarization switching, and domain wall motion can be reduced below the energy differences between the polar orthorhombic phase and these two intermediate phases in pure HfO_2_.

In this work, based on interface engineering, using symmetry analysis and first-principles simulations, we designed a superlattice composed of alternating layers of ferroelectric HfO_2_ and paraelectric CeO_2_ (HCO), vertically aligned along the x-direction. The lateral array of vertically aligned Hf and Ce atoms, which have different ionic sizes and are separated by oxygen layer, activates the highest frequency non-polar distortion (phonon) in the superlattice. Thus, new trilinear phonon–phonon couplings arise, which lead to partial mixing of the ground state monoclinic phase of HfO_2_ with its ferroelectric orthorhombic phase. This mixing in the superlattice leads to a remarkable decrease in the energy difference between its optimized polar orthorhombic and the switching-transition (cubic/tetragonal) phases relative to the difference in bulk HfO_2_. Reducing energy difference between the polar and transition phases is expected to reduce the coercive field (E_c_). In contrast to conventional ferroelectrics, whose polarization decreases with a decrease in E_c_^26^, we discovered that all phonon modes in the polar phase completely survive without suppressing its bulk polarization, and the scale-free nature of ferroelectricity. Thus, the energy barrier for polarization switching in domain wall motion (uniform switching via intermediate tetragonal phase) reduces to 0.57 eV (0.14 eV/u.c.) which is approximately 60% lower than that of HfO_2_. Moreover, as the superlattice in ferroelectrics are known to improve the endurance^27^, the endurance in HCO superlattice may improve relative to its bulk counterpart. Our first-principles findings also validate that HCO exhibits a superior potential for facile polarization switching in comparison to a similar HfO_2_/ZrO_2_ (HZO) superlattice. Moreover, both HfO_2_ and CeO_2_ have already been integrated into silicon technology, and such superlattices can be favorable for experimental growth, as demonstrated in VANs such as La_0.7_Ca_0.3_MnO_3_/MgO^28^ and CoFeO_4_/BaTiO_3_^29^. We believe that our results will open new avenues for the development of low-voltage and high-performance ferroelectric-based devices, such as ferroelectric random-access memory (FeRAM) and ferroelectric field-effect transistor (FeFET).

## Results and discussion

**Figure 1:**
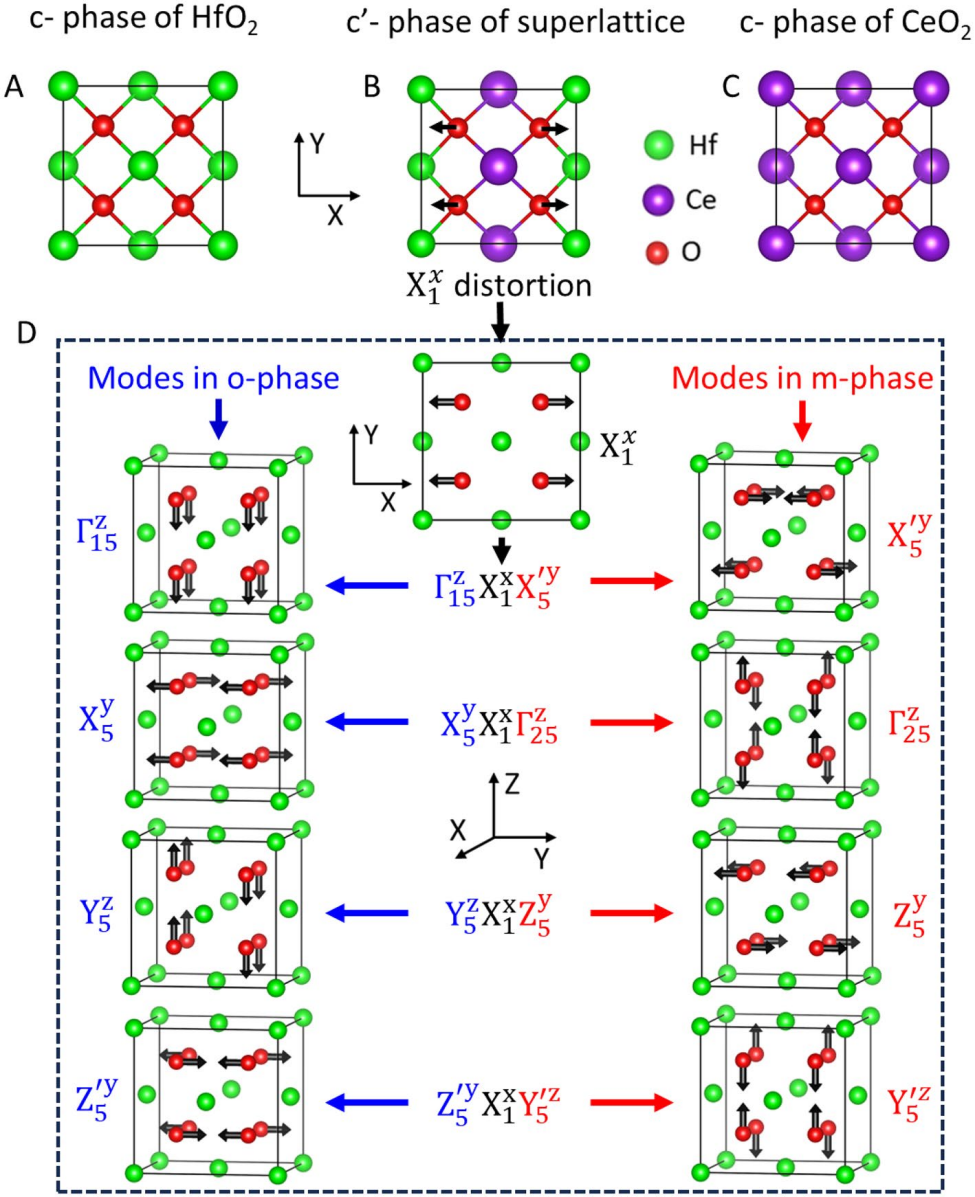
Phonon modes mixing of orthorhombic and monoclinic phases via their trilinear coupling with ${X}_{1}^{x}$ phonon. Atomic structure of cubic phases of HfO_2_ (**A**), superlattice (**B**), and CeO_2_ (**C**). (**D**) The ${X}_{1}^{x}$ mode (at the top) shows trilinear couplings between the phonon modes of the reference cubic phase, which condense in the orthorhombic (${\Gamma }_{15}^{z},{X}_{5}^{y},{Y}_{5}^{z}$, and ${Z}_{5}^{{\prime}y}$, blue in the left column) and monoclinic (${X}_{5}^{{\prime}y},{\Gamma }_{25}^{z},{Z}_{5}^{y}$, and ${Y}_{5}^{{\prime}z}$, red in the right column) phases. All the phonon modes are visualized in the 12-atom unit cell of high-symmetry cubic structure of HfO_2_.

**Figure 2:**
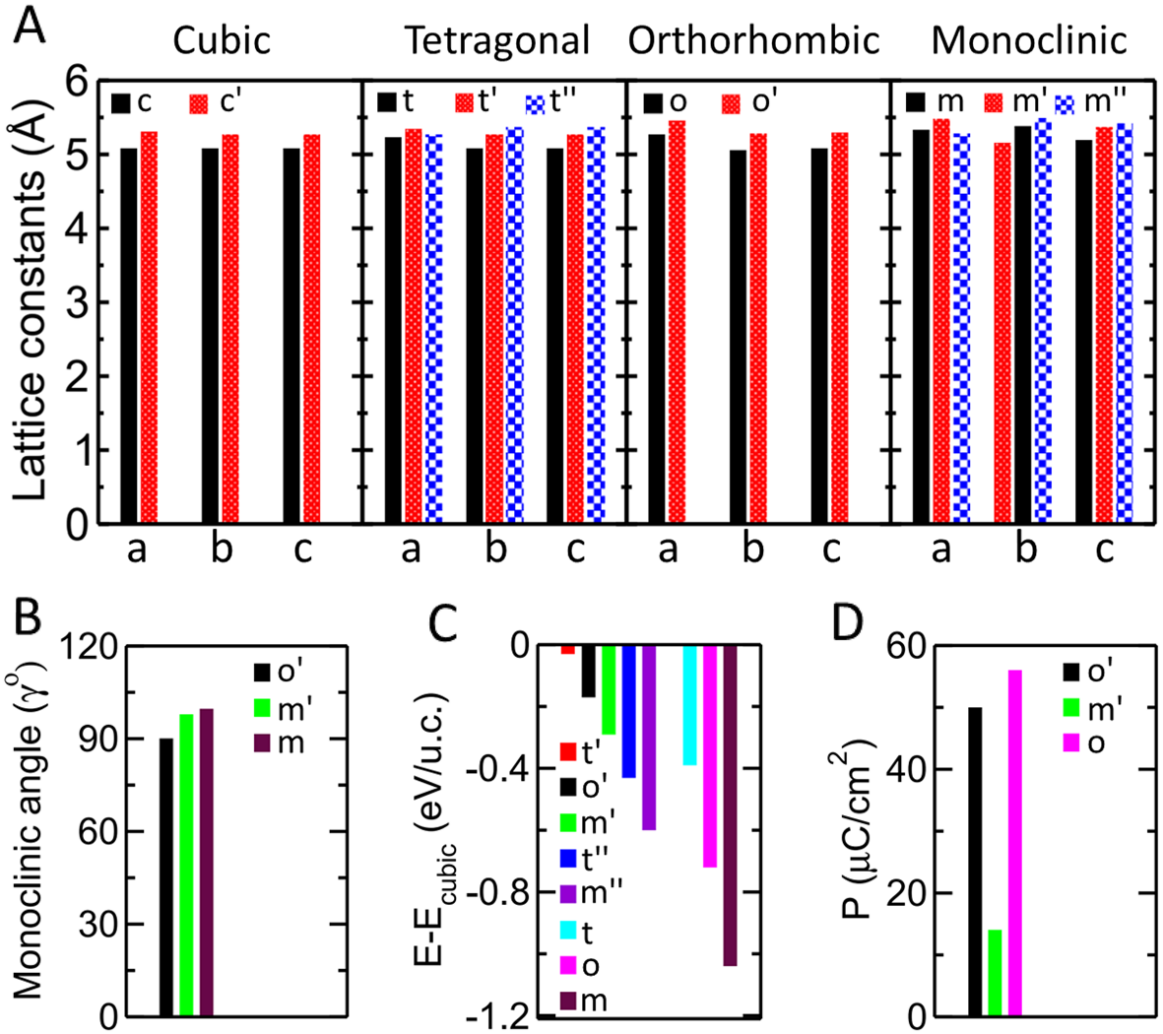
Structural parameters, energies and Polarizations estimated by first-principles calculations. (**A**) The bar chart shows the optimized lattice constants of cubic (c and c'), tetragonal (t, t' and t''), orthorhombic (o and o') and monoclinic (m, m' and m'') structures of bulk HfO_2_ and HCO superlattice. The x-axis represents the lattice constants a, b and c while y-axis denotes their amplitude in angstrom. (**B**) The monoclinic angle induced in the optimized o'-phase (black bar) and of m'-phase (green bar) of HCO superlattice, and m-phase of bulk HfO_2_. (**C**) Total energies of different phases of HfO2 and HCO superlattice relative to their respective optimized cubic structures. (**D**) Polarization of polar o'-phase (black) and m'-phase (green) of superlattice (black), and o-phase of bulk HfO_2_. The notations of different structures with prime and without prime represent structures of HCO superlattice and bulk HfO_2_, respectively.

We designed a vertically aligned superlattice composed of alternating layers of two different fluorite oxides, namely ferroelectric HfO_2_ and paraelectric CeO_2_, in the x-direction. To determine the structural features of the superlattice, we began our analysis with its structural optimization in the reference cubic $${F}_{m\overline{3}m}$$ (c-phase) structure (Fig. 1A, Fig. S1A), which stabilizes above 2870 K^30,31^ in bulk HfO_2_. The ionic size of Ce^4+^ (ionic radius ($${r}_{ion})=1.07$$Å)^32^ is larger than that of Hf^4+^ ($${r}_{ion}$$ = 0.71 Å)^32^; hence, in the optimized cubic structure (c'-phase), the lattice parameters significantly increased relative to the c-phase of HfO_2_ (Fig. 2A and Table S1), and the oxygen atoms in the yz-plane displaced away from Ce and towards Hf (Fig. 1B, Fig. S1). Thus, despite being the highest frequency phonon mode (Fig. S2A and B) in the c-phases of HfO_2_ and CeO_2_ (Fig. 1C), an unsuppressible non-polar $${X}_{1}^{x}$$ phonon mode appears in the c'-phase of HCO, which involves opposite x displacements of oxygen atoms in the alternate yz-planes (Fig. 1D, the atomic displacement of the mode is visualized in the reference $${F}_{m\overline{3}m}$$ phase of HfO_2_).

Through the ${X}_{1}^{x}$ phonon mode, we found a unique relationship between the polar orthorhombic $Pca{2}_{1}$ (o-phase) and non-polar monoclinic $P{2}_{1}/c$ (m-phase) phases (Fig. S3B and C), which stabilize separately in HfO_2_^31,33^. This symmetry-driven relationship between the both phases is crucial in considerably reducing the polarization-switching barrier in the HCO superlattice (discussed later in this manuscript). A total of eight phonon modes, six oxygen-modes (${X}_{2}^{{\prime}x}$, ${\Gamma }_{15}^{z}$, ${X}_{5}^{y}$, ${Y}_{5}^{z}$, ${Z}_{5}^{x}$, and ${Z}_{5}^{{\prime}y}$) and two Hf-modes ${Z}_{5}^{{\prime}x}$, and ${Y}_{3}^{{\prime}y}$ are involved in the phase transition from the cubic to o-phase, while six oxygen-modes (${X}_{2}^{{\prime}x}$, ${\Gamma }_{25}^{z}$, ${Z}_{5}^{x}$, ${Z}_{5}^{y}$, ${X}_{5}^{{\prime}y}$, and ${Y}_{5}^{{\prime}z}$) and three Hf-modes (${X}_{5}^{{\prime}z}$, ${Y}_{5}^{{\prime}x}$, and ${Y}_{3}^{{\prime}y}$), total nine modes, contribute in the phase transition from cubic to m-phase (Fig. 1D and Fig. S4). The phonon spectrum of the c-phase of HfO_2_ shows dominant instability in ${X}_{2}^{{\prime}x}$ mode (Fig. S2A), which involves antiparallel displacements along the x-direction of neighboring oxygen atoms (Fig. S2D). The unstable ${X}_{2}^{{\prime}x}$ leads the transformation from cubic to orthorhombic and monoclinic phases as a primary order parameter with their respective secondary order-parameters (phonon modes), which are quite stable in HfO_2_ (Fig. S2A). Using symmetry analysis and group theory, we found that four oxygen-modes ${\Gamma }_{15}^{z}$, ${X}_{5}^{y}$, ${Y}_{5}^{z}$, and ${Z}_{5}^{{\prime}y}$ of the o-phase show trilinear phonon–phonon coupling respectively with four oxygen-modes ${X}_{5}^{{\prime}y}$, ${\Gamma }_{25}^{y}$, ${Z}_{5}^{y}$ and ${Y}_{5}^{{\prime}z}$ of the m-phase, via the ${X}_{1}^{x}$ mode (Fig. 1D). Similarly, the Hf-modes ${Z}_{5}^{{\prime}x}$ and ${Y}_{5}^{{\prime}x}$ of the o- and m-phases, respectively, show trilinear coupling with the ${X}_{1}^{x}$ mode (Fig. S4A). We also found trilinear coupling between the oxygen- and Hf-modes of the o- and m-phases: the ${Z}_{5}^{{\prime}x}$, ${Z}_{5}^{{\prime}y}$, and ${\Gamma }_{15}^{z}$ modes of the o-phase respectively couple with the ${Y}_{5}^{{\prime}x}$, ${Y}_{5}^{{\prime}z}$, and ${X}_{5}^{{\prime}z}$ modes of the m-phase, mediated by the ${X}_{1}^{x}$ mode (Fig. S4A). While two oxygen-modes ${X}_{2}^{{\prime}x}$ and ${Z}_{5}^{x}$, one Hf-mode ${Y}_{3}^{{\prime}y}$ (Fig. S4B–D), which do not show trilinear coupling with any other existing modes via the ${X}_{1}^{x}$ mode, are common in both phases. Thus, the ${X}_{1}^{x}$ mode distortion can induce phonon components of the ground state m-phase into the polar o-phase and play a crucial role in their mutual mixing.

To characterize the unusual mixing of the orthorhombic and monoclinic structures, we use group theory to obtain all the trilinear coupling terms among their respective oxygen phonon modes. In this process, we take the high symmetry cubic phase as a basis and write the trilinear coupling terms of as follows,

1 $${H}_{ph}^{3}= {b}_{1}{Q}_{{\Gamma }_{15}^{z}}{Q}_{{X}_{1}^{x}}{Q}_{{X}_{5}^{{\prime}y}}+{b}_{2}{Q}_{{X}_{5}^{y}}{Q}_{{X}_{1}^{x}}{Q}_{{\Gamma }_{25}^{z}}+{b}_{3}{Q}_{{Y}_{5}^{z}}{Q}_{{X}_{1}^{x}}{Q}_{{Z}_{5}^{y}}+ {b}_{4}{Q}_{{Z}_{5}^{{\prime}y}}{Q}_{{X}_{1}^{x}}{Q}_{{Y}_{5}^{{\prime}z} } +{ b}_{5}{Q}_{{X}_{2}^{{\prime}x}}{Q}_{{\Gamma }_{15}^{z}}{Q}_{{X}_{5}^{y}}+{b}_{6}{Q}_{{X}_{2}^{{\prime}x}}{Q}_{{Y}_{5}^{z}}{Q}_{{Z}_{5}^{{\prime}y}} +{b}_{7}{Q}_{{\Gamma }_{15}^{z}}{Q}_{{Z}_{5}^{x}}{Q}_{{Z}_{5}^{{\prime}y}}+ {b}_{8}{Q}_{{X}_{5}^{y}}{Q}_{{Y}_{5}^{z}}{Q}_{{Z}_{5}^{x}} +{b}_{9}{Q}_{{X}_{2}^{{\prime}x}}{Q}_{{\Gamma }_{25}^{z}}{Q}_{{X}_{5}^{{\prime}y}}+{b}_{10}{Q}_{{X}_{2}^{{\prime}x}}{Q}_{{Y}_{5}^{{\prime}z}}{Q}_{{Z}_{5}^{y}}+{b}_{11}{Q}_{{\Gamma }_{25}^{z}}{Q}_{{Z}_{5}^{x}}{Q}_{{Z}_{5}^{y}}+ {b}_{12}{Q}_{{X}_{5}^{{\prime}y}}{Q}_{{Y}_{5}^{{\prime}z}}{Q}_{{Z}_{5 }^{x}} + {b}_{13}{Q}_{{Y}_{5}^{z}}{Q}_{{X}_{5}^{{\prime}y}}{Q}_{{Z}_{5}^{{\prime}x}}$$

where ${H}_{ph}^{3}$ include trilinear couplings, ${b}_{i}$ denote their respective coefficients. $Q$ denotes the amplitude of the subscripted phonon modes. We note that the first four trilinear coupling terms in Eq. (1) with coefficients ${b}_{1},{b}_{2},{b}_{3}$, and ${b}_{4}$, denote the coupling between the orthorhombic and monoclinic modes via the ${X}_{1}^{x}$ mode and are solely responsible for the mixing of these two structures. The terms with coefficients ${b}_{5},{b}_{6},{b}_{7}$, and ${b}_{8}$ (${b}_{9},{b}_{10}={b}_{6},{b}_{11}$ and ${b}_{12}$) indicate trilinear coupling among the orthorhombic (monoclinic) modes. The additional non-polar ${Z}_{5}^{{\prime}x}$ mode mediates the trilinear coupling, the last term with coefficient ${b}_{13} (={b}_{12})$ in Eq. (1), between two non-polar zone boundary modes ${X}_{5}^{{\prime}y}$ and ${Y}_{5}^{z}$ of the monoclinic and orthorhombic structures, respectively. Furthermore, these trilinear terms are also quite important to identify the symmetry equivalent invariants of ferroelectric phases and their domain walls as we will discuss later in this manuscript. To use ${H}_{ph}^{3}$ for other analysis, one can estimate the coefficients ${b}_{i}$ using frozen phonon calculations of their respective modes in DFT simulations. However, it's important to note that this task is outside the scope of this work.

**Figure 3:**
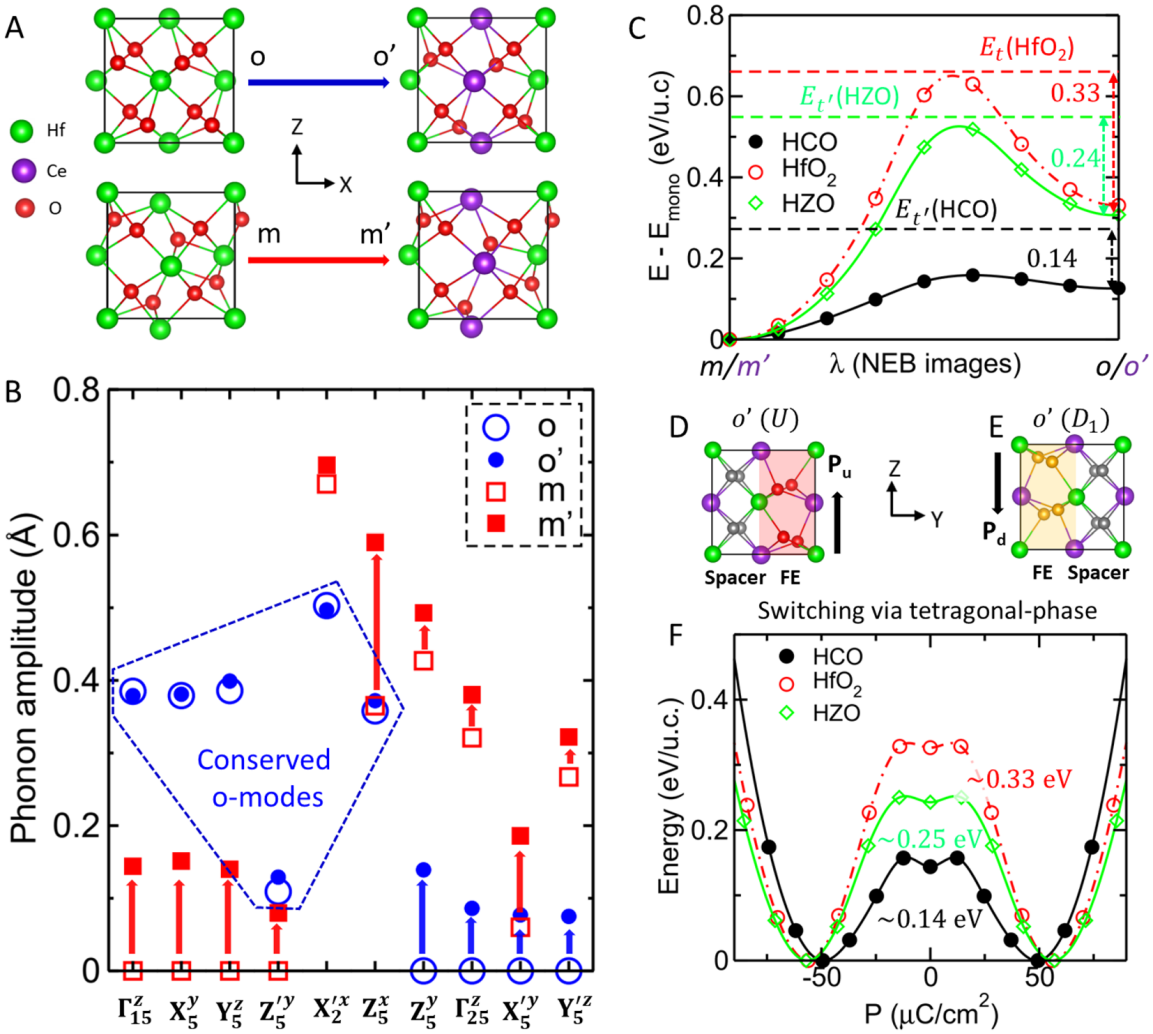
Phonon engineering to preserve the modes in ferroelectric against monoclinic non-polar modes inclusion and polarization switching. (**A**) Atomic structures of the orthorhombic and monoclinic phases of HfO_2_ and HCO superlattice. (**B**) Amplitude of the phonon modes that are condensed in the o-phase (blue open circle), o'-phase (filled blue circle), m-phase (red open square), and m'-phase (red filled square). The blue and red arrows show the increase in phonon amplitude in the o'- and m'-phases relative to their counterparts in HfO_2_. The blue dashed-line box contains the conserved modes of the o-phase in the o'-phase. (**C**) Energy barrier estimated by variable cell NEB calculations along the transition path between the monoclinic and orthorhombic phases of the superlattice (black filed circles) and HfO_2_ (red open circles). The dashed black, red and green lines indicate the energies of the tetragonal phases of the HCO, HfO_2_ and HZO, respectively, while $\lambda$ represents the transition co-ordinate between their monoclinic and orthorhombic phases. Atomic structures of the up-polarized (**D**) and down-polarized (**E**) o'-phases of HCO. The silver oxygen atoms belong to the spacer layer whereas the red and yellow oxygen atoms belong to the up-polarized and down-polarized ferroelectric layers, respectively. (**F**) Energy landscape along the paths of switching homogeneous polarization via an intermediate tetragonal phase in the HCO superlattice (filled black circles), HfO_2_ (open red circles) and HZO superlattice (open green diamonds).

To realize the mixing of the o^−^ and m-phases, we optimized both the phases individually in the HCO superlattice and decomposed their atomic position distortion into phonon modes of reference c-phase (Eq. S1 in SI) using the scheme introduced by K. M. Rabe et al.^34^. In the optimized orthorhombic (o'-phase) and monoclinic (m'-phase) phases (Fig. 3A, Fig. S5A, B), the amplitudes of all phonon modes of m- and o-phases, respectively, emerged with 25% to 40% of their original values (Fig. 3B and Table S2). The appearance of new phonon modes of the o-phase in the m'-phase also enhances its original modes. In contrast, the original phonon modes remain conserved (see dashed blue line box in Fig. 3B) in the o'-phase despite the emergence of new m-phase phonon modes. Interestingly, the amplitudes of the ${\Gamma }_{15}^{z}$ (0.379 Å) and ${Y}_{5}^{z}$ (0.399 Å) modes in o'-phase are almost same that make a dipolar partitioning into two types of alternate dead and active layers in the orthorhombic phase^22^. Thus, the spacer and ferroelectric active layers remain perfectly placed in the o'-phase (Fig. 3D), and its estimated polarization of 50 $\mu C/c{m}^{2}$ remains similar to that of bulk HfO_2_ of 56 $\mu C/c{m}^{2}$ (Fig. 2D). As a result of this unusual mixing, the energy difference between the o'- and m'-phases reduces to 0.12 eV/u.c. from 0.27 eV/u.c. in bulk HfO_2_ (Fig. 2C and Table S1). However, they remain well separated by an energy barrier of 0.03 eV/u.c., which is 0.32 eV/u.c. for HfO_2_ (Fig. 3C). The lattice parameters and energies of the orthorhombic and monoclinic phases of the superlattice and HfO_2_ are listed in (Fig. 2A–C and Table S1). As a result of unusual mixing of polar and nonpolar phases, a small polarization of 14 $\mu C/c{m}^{2}$ also induces in the m'-phase (Fig. 2D).

We now relaxed the HCO superlattice in the tetragonal $P{4}_{2}/mnc$ (t-phase) of HfO_2_ (Fig. S3A), which induces due to the condensation of the most unstable ${X}_{2}^{{\prime}x}$ mode in the c-phase and an intermediate phase appearing during polarization switching in the o-phase through a lower energy barrier^24,25^. We found that in the optimized tetragonal (t'-phase) structure (Fig. S6A), the ${X}_{1}^{x}$ mode appears as well, and its energy difference from the c'-phase reduces to 0.03 eV/u.c., which is 0.39 eV/u.c. for HfO_2_ (Fig. 2C). This is due to the instability of the ${X}_{2}^{{\prime}x}$ mode in the c'-phase reduces to $\omega =$ − 96 $c{m}^{-1}$ (Fig. S1C) from − 224 $c{m}^{-1}$ in the c-phase of HfO_2_ (Fig. S1A). The instability of ${X}_{2}^{{\prime}x}$ mode reduces because of two reasons: first, the atomic modulation of this mode is in the direction of the superlattice, and second, the same mode is quite stable ($\omega =157 c{m}^{-1}$) in the c-phase of CeO_2_ (Fig. S1B). We note that the most unstable $(\omega =$ − 221 $c{m}^{-1}$) phonon mode (Fig. S2C) in the superlattice is an average of the unstable ${Y}_{2}^{{\prime}y}$ and ${Z}_{2}^{{\prime}z}$ phonon modes (Fig. S2E–G), which remains similar to the c-phase of HfO_2_, despite being the stability of these modes in the c-phase of CeO_2_ (Fig. S2B). This is because the oxygen displacements in these modes are perpendicular to the direction of the superlattice (Fig. S2E–G). The condensation of this most unstable phonon in the c'-phase transforms it into another non-polar tetragonal (t''-phase) structure (Fig. S6B); further condensation of the three oxygen-modes (${\Gamma }_{25}^{y}$, ${X}_{5}^{z}$, and ${Z}_{5}^{{\prime}x}$) and two Hf-modes (${Y}_{3}^{{\prime}y}$ and ${Z}_{5}^{{\prime}y}$) transforms it into another non-polar monoclinic (m''-phase) structure (Fig. S5C) which is the lowest energy phase of the superlattice (Fig. 2C). The lattice parameters and energies of the t''- and m''-phases of the superlattice are listed in Fig. 2A–D and Table S1, and the amplitude of phonon modes which condensed in these structures are listed in Fig. S7.

Strikingly, the emergence of monoclinic modes in the o'-phase of HCO leads to an increase in the energy of the later phase because it is separated by a quite large energy barrier of 0.32 eV/u.c from the former phase in the bulk HfO_2_. This impacts the energy difference between the t'- and o'-phases which reduces to 0.14 eV/u.c. from 0.33 eV/u.c. in bulk HfO_2_. The energy difference between the orthorhombic and tetragonal phases is proportional to the energy barrier of the uniform polarization switching between the up-polarized o'-phase, called U (Fig. 3D), and the down-polarized phase, called D_1_ (Fig. 3E). Thus, the energy barrier of uniform polarization switching through the t'-phase in the HCO superlattice decreases by almost 60% from its bulk value (Fig. 3F). The D_1_-phase is symmetrically and energetically identical to the U-phase; they exchange their spacer and ferroelectric active layers (Fig. 3D and E) because the polar mode reverses its sign, whereas the ${Y}_{5}^{z}$ mode remains unchanged (Table S2). To ensure that the energy function (Eq. (1)) is invariant, five other oxygen modes (${X}_{5}^{y}$, ${Z}_{5}^{x}$, ${\Gamma }_{25}^{y}$, ${X}_{5}^{{\prime}y}$, and ${Z}_{5}^{{\prime}x}$) and three hafnium modes (${Y}_{3}^{y}$, ${X}_{5}^{{\prime}z}$, and ${Z}_{5}^{{\prime}y}$) switch their sign in the D_1_-phase relative to the U-phase (Table S2). In particular, the ${X}_{2}^{{\prime}x}$ and ${X}_{1}^{x}$ modes do not reverse their sign in the D_1_-phase. Thus, the polarization switching between the U- and D_1_-phases passes through the intermediate t'-phase.

For comparative analysis of HCO with a similar vertically aligned ZrO2/HfO2 (HZO) superlattice in the x-direction, we optimized the later superlattice in the c-, t-, o-, m-phases of HfO_2_. Our findings reveal that, in contrast to HCO, the highest frequency ${X}_{1}^{x}$ mode in the optimized c'-, t'-, o'- and m'-phases (Figs. S8 and S9) of HZO induces quite weakly (${Q}_{{X}_{1}^{x}}$ = − 0.013Å to − 0.010Å) (Table S3). This is because the difference in the ionic size between Zr^4+^ (${r}_{ion}=0.780$Å)^32^ and Hf^4+^ (${r}_{ion}=0.710$Å) is quite small compared to Ce^4+^ (${r}_{ion}=1.070$Å). Consequently, the mixing of the phonon modes of m-phase (o-phase) in the optimized o'-phase (m'-phase) of HZO is vanishingly small (Table S3) which plays a crucial role in reducing the energy barrier for polarization switching in the ferroelectric o'-phase. Moreover, in contrast to CeO_2_, the c-phase of ZrO_2_ is dynamically unstable as its ${X}_{2}^{{\prime}x}$ mode shows instability of – 220 $c{m}^{-1}$ (Fig. S10C) which is almost equal to HfO_2_. Thus, the instability of ${X}_{2}^{{\prime}x}$ mode in the HZO superlattice does not reduce and its estimated frequency $\omega$ is − 221 $c{m}^{-1}$ (Fig. S10A). Notably, t-, o- and m-phases individually stabilize in bulk ZrO_2_ (Fig. S11), similar to bulk HfO_2_. Therefore, the lattice parameters and energetics of these optimized phases in HZO show minimal variation, approximating the numerical averages of bulk HfO_2_ and ZrO_2_ (Table S3). The estimated polarization 57.1 $\mu C/c{m}^{2}$ (Table S3) in the optimized o'-phase of HZO also found close to the o-phase of HfO_2_, and this phase is well separated by an energy barrier of 0.21 eV/u.c from the m'-phase (Fig. 3C). We also determined that the energy barrier for polarization switching between the U- and D_1_-phases HZO is 0.25 eV/u.c (Fig. 3F), which is 0.10 eV/u.c higher than for HCO, average of HfO_2_ and ZrO_2_ (Fig. S12). Based on this comparative analysis, we conclude that HZO displays a gradual variation in structural and ferroelectric properties compared to bulk HfO_2_, which are averages of HfO_2_ and ZrO_2_, whereas HCO exhibits drastic deviations from the bulk HfO_2_. Consequently, for our further analysis, our primary focus will be on HCO.

**Figure 4:**
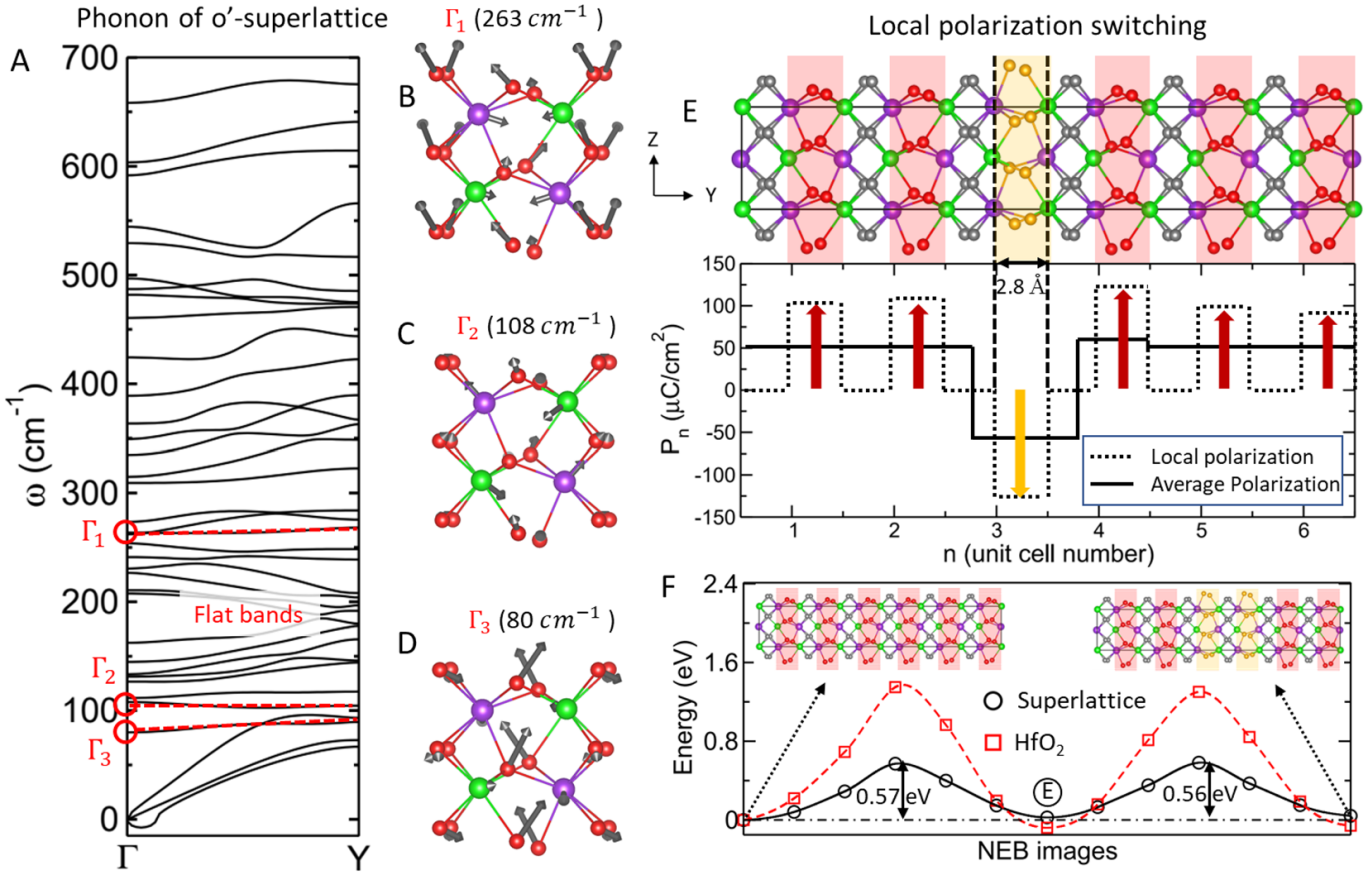
Preserved scale-free ferroelectricity with nearly zero domain wall energy in the superlattice. (**A**) Phonon dispersion of the ferroelectric o'-phase of the superlattice along $\Gamma \to Y$ has three flat phonon bands (highlighted with dashed red line, the deviation in the frequency of the flat band is less than $10 c{m}^{-1}$). (**B**–**D**) Eigenvectors of flat phonon bands at the $\Gamma$-point visualized in terms of atomic displacements in the unit cell of o'-phase. (**E**) Atomic structure of the thinnest possible domain in the superlattice along the y-direction (top), local and average polarization of this domain structure (bottom). (**F**) Energy landscape of domain wall motion along the y-direction in the superlattice, starting from a uniformly polarized structure (top-left corner inset) to a structure with two consecutive opposite polarized layers (top-right corner inset) in the superlattice (black open circles) and HfO_2_ (red open squares).

The phonon dispersion of the o'-phase (Fig. 4A) of HCO shows three flat phonon bands along $\Gamma \to Y$. The $\Gamma$-point phonon of these three flat bands involve atomic displacements of all phonon modes, which transform the high-symmetry cubic phase into the o'-phase (Fig. 4B-4D). These flat bands guarantee scale free ferroelectricity in the o'-phase of the superlattice^22^. However, only two such flat phonon bands are present in HfO_2_ (Fig. S13). We believe that the additional flat phonon band appearing in the o'-phase is a result of trilinear couplings between the orthorhombic and monoclinic modes via the ${X}_{1}^{x}$ mode. To confirm the scale-free nature of ferroelectricity in the o'-phase, we analyzed the 180° domains along the y-direction (Fig. S14) in which equal and opposite polarizations across the domain wall are separated by a spacer layer (which is unlikely in the U/D_1_ domain). The antipolar ${X}_{2}^{{\prime}x}$ mode reverses its sign with ${\Gamma }_{15}^{z}$ mode, four other oxygen modes (${Y}_{5}^{z}$, ${Z}_{5}^{x}$, ${Z}_{5}^{y}$, and ${X}_{5}^{{\prime}y}$) and one hafnium mode (${X}_{5}^{{\prime}z}$) (Table S2) that keep the down polarized phase (D_2_-phase) symmetry invariant (Eq. (1)). We found that the polarization at the domain wall was not suppressed in the U/D_2_ domains, and the polarization remained almost equal to its bulk value across the domain wall (Fig. S14), which makes the domain walls very sharp. These unusual domain walls further confirm the scale-free ferroelectricity in the o'-phase of HCO superlattice. The estimated domain wall energy was weakly positive (12.5 mJ/m^2^), and consequently, the domain wall structure was comparably stable with its bulk structure, in contrast to conventional perovskites^35^.

We estimated the energy barrier of 0.36 eV/u.c. in the homogeneous polarization switching between U and D_2_ phases of HCO where the reversal of polarization passes through the cubic-like phase. This barrier is reduced by more than 2.8 times relative to bulk HfO_2_, which is 1.02 eV/u.c. (Fig. S15). This is because the energy difference between the c'- and o'-phases decreases considerably compared to their counterparts in HfO_2_ (Fig. 2C). We estimated the energy barrier and domain wall energy of a locally switched polarization of a half-unit-cell-wide polar domain positioned between two spacer layers (Fig. 4E). The 2.8 Å wide polar layer reverses its polarization with an energy barrier of 0.57 eV (Fig. 4F) without suppressing its bulk value; the energy barrier is over 2.3 times (~ 60%) smaller than that of the HfO_2_ counterpart (1.34 eV)^22^. Other unconventional pathways^36^, such as oxygen passing through Hf and Ce planes, may further reduce the energy barriers. The domain wall energy is weakly positive (7.2 mJ/m^2^), so the domain wall structure is energetically comparable to its bulk structure. Further polarization reversal in the adjacent polar layer (Fig. 4F, inset) also passes through a similar energy barrier of 0.56 eV. We note that all domain wall structures are energetically comparable to the bulk structure; this indicates a weak interaction between the domain walls, an intrinsic characteristic of scale-free ferroelectricity.

In summary, we discovered intrinsic phonon couplings in vertically aligned superlattices that result from the differences between the sizes of the cations in adjacent unit cells of two different metal oxides. The results of our interface engineering scheme in such superlattices provide fundamental physics insights into phase mixing, fundamentally improving material properties. Based on these results, we have revealed the origin of the reduced coercive field while retaining the original strong bulk polarization and proposed a commercialization-ready Si-compatible material (HfO_2_/CeO_2_) which also exhibits a superior potential for facile polarization switching in comparison to a similar HfO_2_/ZrO_2_ superlattice. In the HCO vertical superlattice, stable and independently switchable polar domains can form even at a half-unit cell thickness of ~ 2.8 Å. Hence, our results can have immediate impact to the realization of highly dense memory devices with bulk-strong polarization and remarkably low operational voltages. In addition, this scheme represents a significant advancement in addressing the challenge to reduce the coercive field without decreasing the bulk strong polarization of HfO_2_, opening new avenues for designing next-generation memory devices with significant technological implications.

## Methods

The first-principles calculations in our work were conducted using density functional theory (DFT) based on a plane-wave pseudopotential scheme, as implemented in the Vienna Ab-initio Simulation Package (VASP)^37–39^. We used the projector augmented wave (PAW)^40^ pseudopotential with a generalized gradient approximation (GGA) and Perdew Burke Ernzerhof (PBE)^41^ form of the exchange–correlation energy functional, and $5{s}^{2}5{p}^{6}6{s}^{2}5{d}^{2}$ electronic states of Hf, $4{s}^{2}4{p}^{6}5{s}^{2}6{d}^{2}$ states of Zr, $4{f}^{1}5{s}^{2}5{p}^{6}5{d}^{1}6{s}^{2}$ states of Ce, and 2 ${s}^{2}2{p}^{4}$ states of O as the valence states. The energy cutoff used to truncate the plane-wave basis sets representing the Kohn–Sham wave functions were set to 500 eV. In self-consistent Kohn–Sham calculations with conventional unit cells, Brillouin zone integrations were sampled on uniform meshes, using Monkhorst–Pack (MP)^42^ method, of 8 × 8 × 8 and 8 × 2 × 8 for conventional unit cell and domain wall structures in a 1 × 6 × 1 supercell, respectively. In the structural optimization, the convergence criteria for total energy were set to 10^−7^ eV, and the convergence criterion for total forces was set to 10^−3^ eV/Å. We used the density functional perturbation theory (DFPT) in VASP to determine the lattice-dynamical properties of the superlattice, HfO_2_, and CeO_2_. All atomic structures and phonon mode visualizations in terms of atomic displacements presented in this manuscript were drawn in VESTA^43^; the graphs were plotted using Xmgrace codes.

### Supplementary Information

Supplementary Information.

## Data Availability

All data are available in the main text and supplementary materials.
