# Supplementary material for: Interface engineering for facile switching of bulk-strong polarization in Si-compatible vertical superlattices
Source: Sci Rep. 2024 Mar 21;14:6811. doi: 10.1038/s41598-024-56997-0 (PMC10958034; doi:10.1038/s41598-024-56997-0)
Supplement: Supplementary file 1 — Supplementary Information. [file 41598_2024_56997_MOESM1_ESM.pdf]

Supplementary information

Interface engineering for facile switching of bulk-strong polarization in Si-compatible vertical superlattices

Pawan Kumar[1] and Jun Hee Lee[*,1,2]

[1]Department of Energy Engineering, School of Energy and Chemical Engineering, Ulsan National Institute of Science and Technology (UNIST), Ulsan 44919, Republic of Korea

[2]Graduate School of Semiconductor Materials and Devices Engineering, Ulsan National Institute of Science and Technology (UNIST), Ulsan, 44919, Republic of Korea

[*] junhee@unist.ac.kr

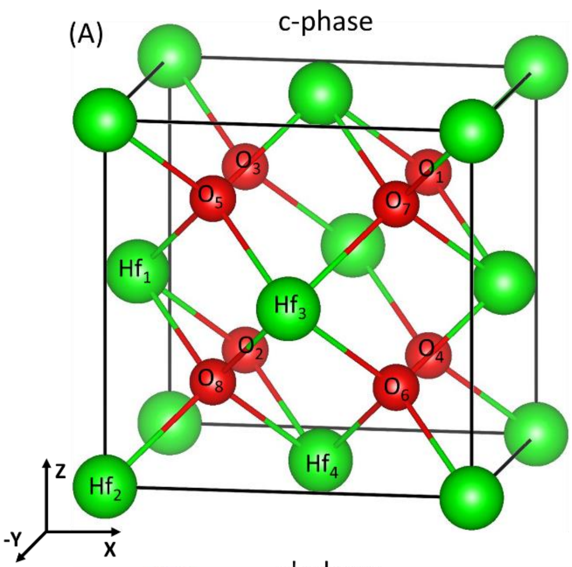

(B)

Atoms	c-phase		
	x	y	z
Hf_1	0.0000	0.5000	0.5000
Hf_2	0.0000	0.0000	0.0000
Hf_3	0.5000	0.0000	0.5000
Hf_4	0.5000	0.5000	0.0000
O_1	0.7500	0.7500	0.7500
O_2	0.2500	0.7500	0.2500
O_3	0.2500	0.7500	0.7500
O_4	0.7500	0.7500	0.2500
O_5	0.2500	0.2500	0.7500
O_6	0.7500	0.2500	0.2500
O_7	0.7500	0.2500	0.7500
O_8	0.2500	0.2500	0.2500

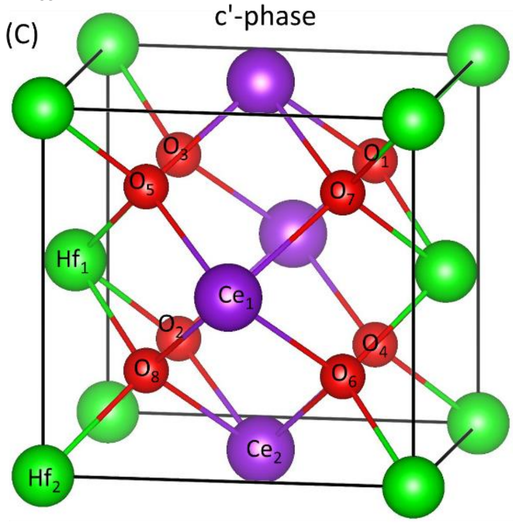

(D)

Atoms	c'-phase		
	x	y	z
Hf_1	0.0000	0.5000	0.5000
Hf_2	0.0000	0.0000	0.0000
Ce_1	0.5000	0.0000	0.5000
Ce_2	0.5000	0.5000	0.0000
O_1	0.7653	0.7500	0.7500
O_2	0.2347	0.7500	0.2500
O_3	0.2347	0.7500	0.7500
O_4	0.7653	0.7500	0.2500
O_5	0.2347	0.2500	0.7500
O_6	0.7643	0.2500	0.2500
O_7	0.7643	0.2500	0.7500
O_8	0.2347	0.2500	0.2500

Figure S1 | Cubic phases of HfO_2 and HCO superlattice. (A) Visualization of atomic structure of c-phase of HfO_2 in its conventional unit cell of 12 atoms, and (B) its atomic positions in the crystal coordinates. (C) Visualization of atomic structure of c'-phase of superlattice, and its atomic positions in the crystal coordinates (D). In c'-phase, Hf_3 and Hf_4 of c-phase of HfO_2 are replaced by Ce_1 and Ce_2, respectively.

Table S1. Structural parameters and energies estimated by first-principles calculations. Structural parameters and total energies of the cubic, tetragonal, orthorhombic, and monoclinic structures of the superlattice and HfO_2 relative to their cubic structures and their polarizations.

Structures	a (Å)	b (Å)	c (Å)	γ° (β° for m'')	$E\text{-}E_{cubic}$ ($eV/u.c.$)	Polarization ($\mu C/cm^2$)
c'	5.298	5.258	5.258	90	0	0
c	5.079	5.079	5.079	90	0	0
t'	5.341	5.259	5.259	90	-0.03	0
t	5.227	5.082	5.082	90	-0.39	0
t''	5.266	5.366	5.365	90	-0.43	0
o'	5.456	5.277	5.292	90.03	-0.17	50
o	5.273	5.055	5.085	90	-0.72	56
m'	5.481	5.383	5.369	97.82	-0.29	14
m	5.329	5.147	5.199	99.68	-1.04	0
m''	5.279	5.494	5.419	95.91	-0.60	0

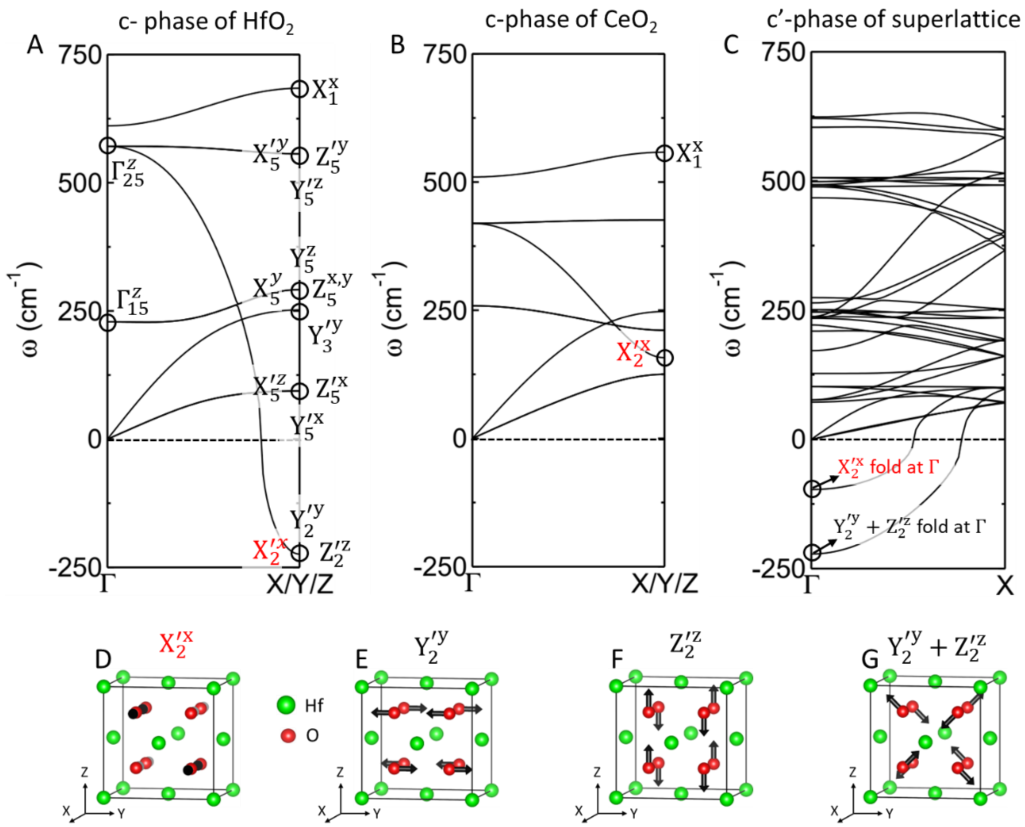

Figure S2 | Phonon spectra of cubic structures of HfO_2, CeO_2 and HCO superlattice. (A-C) Phonon spectra of optimized cubic phases of HfO_2 (primitive unit cell with 3 atoms: 1 hafnium and 2 oxygen), CeO_2 (primitive unit cell) and superlattice (conventional unit cell with 12 atoms: 4 hafnium and 8 oxygen), respectively. The imaginary frequencies ($\omega^2 < 0$) depicted with negative sign in phonon spectra of HfO_2 and superlattice, which make them dynamically unstable at low temperature in their respective cubic phases, while phonon spectrum of CeO_2 does not show any imaginary frequencies, and hance this is dynamically stable in its cubic phase. Phonon modes which are involved in the cubic to tetragonal, orthorhombic and monoclinic phase transformations are shown in the phonon spectrum of HfO_2. (D-G) Depict atomic displacements (black arrows) of $X_2^{\prime x}$, $Y_2^{\prime y}$, $Z_2^{\prime z}$, and $Y_2^{\prime y} + Z_2^{\prime z}$ modes which are unstable in phonon spectra of HfO_2 and the superlattice.

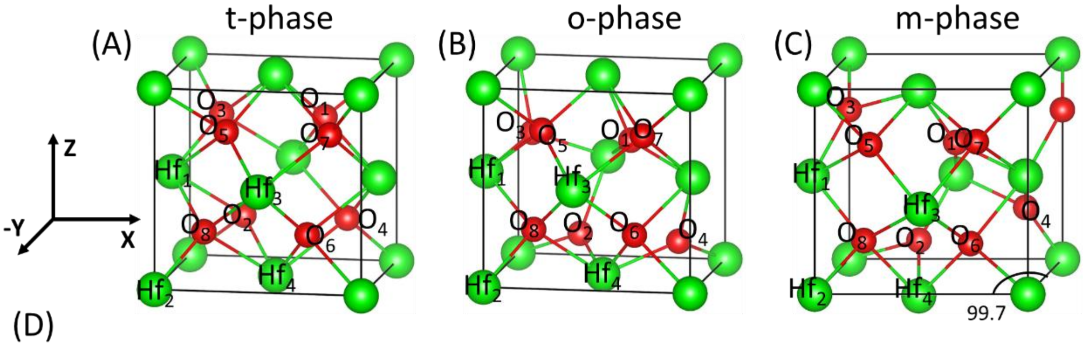

(D)

Atoms	t-phase			o-phase			m-phase		
	x	y	z	x	y	z	x	y	z
Hf_1	0.0000	0.5000	0.5000	-0.0660	0.4668	0.5000	-0.0844	0.4481	0.4998
Hf_2	0.0000	0.0000	0.0000	0.0000	0.0000	0.0000	0.0002	0.0000	-0.0002
Hf_3	0.5000	0.0000	0.5000	0.4340	0.0000	0.5000	0.5002	0.0000	0.4148
Hf_4	0.5000	0.5000	0.0000	0.5000	0.4667	0.0000	0.4156	0.4480	-0.085
O_1	0.6963	0.7500	0.7500	0.6015	0.6651	0.6427	0.5551	0.6561	0.6272
O_2	0.3037	0.7500	0.2500	0.3324	0.8016	0.1427	0.3607	0.7920	0.1272
O_3	0.1963	0.7500	0.7500	0.1015	0.8061	0.6427	0.0551	0.6561	0.7874
O_4	0.8037	0.7500	0.2500	0.8324	0.6651	0.1427	0.8607	0.7920	0.2874
O_5	0.3037	0.2500	0.7500	0.2364	0.3711	0.7501	0.2301	0.1728	0.7000
O_6	0.6963	0.2500	0.2500	0.6976	0.1956	0.2501	0.6857	0.2752	0.2000
O_7	0.8037	0.2500	0.7500	0.7364	0.1956	0.7501	0.7301	0.1728	0.7147
O_8	0.1963	0.2500	0.2500	0.1976	0.2711	0.2501	0.1857	0.2752	0.2147

Figure S3 | Low-symmetry phases of HfO_2. Visualization of atomic structures of tetragonal (t-phase) (A), orthorhombic (B) and monoclinic (C) phases of HfO_2. (D) atomic positions in the crystal coordinates of these structures.

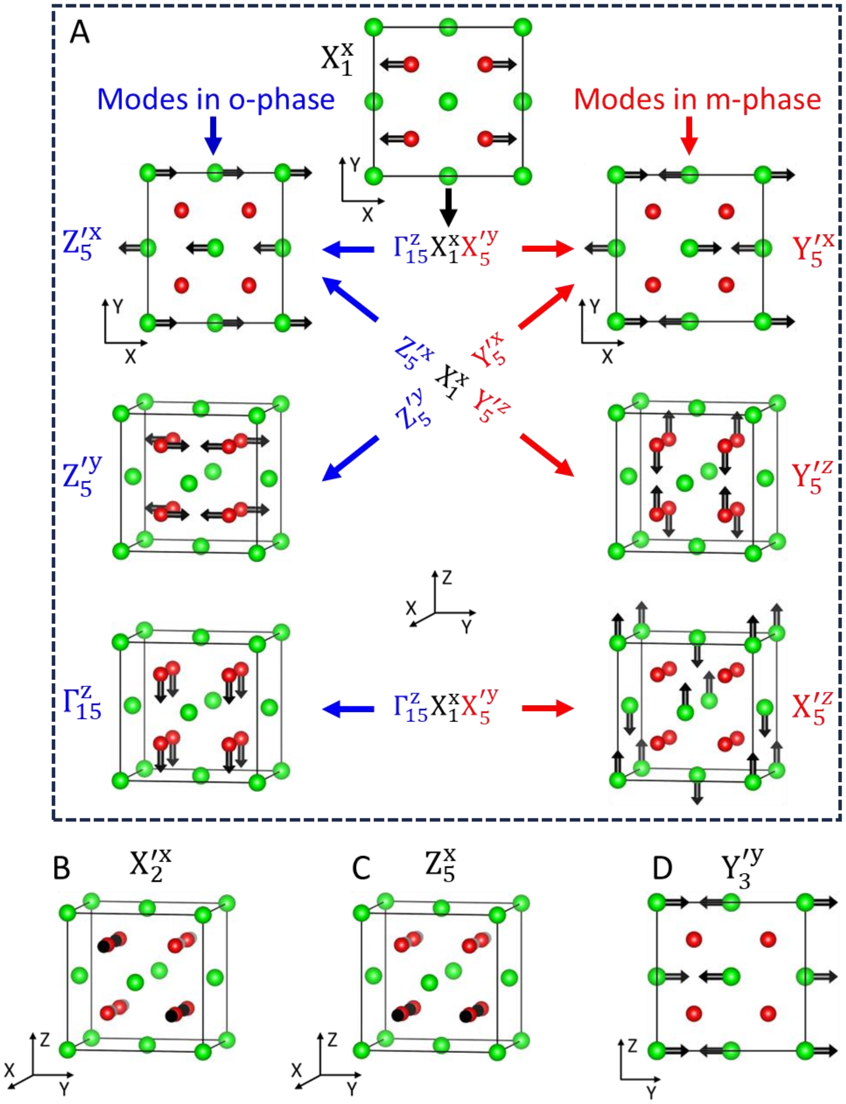

Figure S4 | Phonon modes mixing of orthorhombic and monoclinic phases via their trilinear coupling with X_1^x phonon. (A) The X_1^x mode (at the top) shows trilinear couplings between phonon modes of reference cubic phase which condense in orthorhombic ($Z_5^{\prime x}$, $Z_5^{\prime y}$, and Γ_{15}^z, blue color in left column) and monoclinic ($Y_5^{\prime x}$, $Y_5^{\prime z}$, and $X_5^{\prime z}$, red color in right column) phases. The $X_2^{\prime x}$ (B), Z_5^x (C), and $Y_3^{\prime y}$ (D) do not show trilinear coupling with any existing phonon modes in orthorhombic and monoclinic phases via X_1^x. All the phonon modes are visualized in the 12 atoms unit cell of high-symmetry cubic structure of HfO_2.

Method for estimation of phonon amplitude

To determine the phonon modes amplitudes, we have used the scheme proposed by K. M. Rabe et.al[1]. In this scheme, we first calculated the atomic position differences $\vec{d}$ between c-phase (reference structure) and optimized phases of HfO_2, ZrO_2 and different superlattices. Now, we calculated the phonon mode amplitude of each mode by the inner product between its eigenvector $\vec{u}$ and $\vec{d}$ as follows,

$$\textit{Phonon mode amplitude mode (Å)} = \frac{1}{2}\sum_{i=1}^{12} \vec{u}_i \cdot \vec{d}_i, \quad \text{(S1)}$$

where, $\vec{u}_i$ and $\vec{d}_i$ are the normalized component of mode's eigenvector and displacement from high symmetry position of ith atoms in the conventional unit cell (12 atoms: 4 Hf and 8 O), respectively. Since each conventional unit cell has total of eight oxygen atoms, therefore for oxygen modes the non-zero normalized component of $\vec{u}_i$ is $\frac{1}{\sqrt{8}}$. While it has four hafnium atoms, thus, the non-zero normalized component of $\vec{u}_i$ is $\frac{1}{2}$ in Hf-mode. The factor of $\frac{1}{2}$ in Eq. (S1) further renormalizes the mode's amplitude with respect to primitive unit cell (3 atoms: 1 Hf and 2 O) of high symmetry c-phase. The amplitudes of phonon modes in different phases of superlattice and bulk HfO_2 are listed in Table S2 and S3. The amplitudes of phonon modes are quite critical to estimate their contribution in the atomic distortion of different phases of HfO_2 structures in the superlattice, and help to determine different switching pathways and their energetics. The eigenvectors of each phonon mode are visualized in the conventional unit cell of c-phase of HfO_2 in Figure 1D in manuscript, Figures S2, S4 and S7.

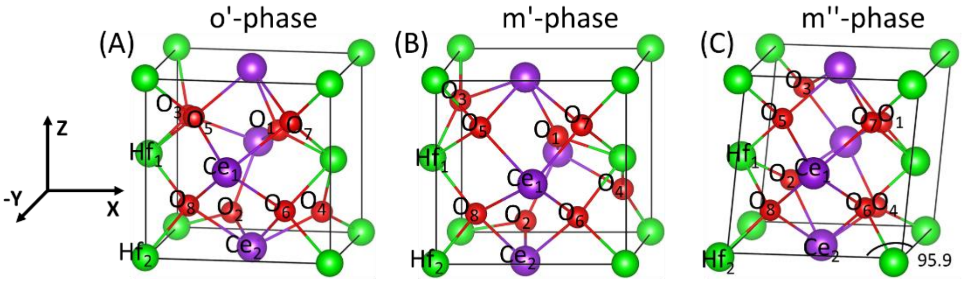

(D)

Atoms	o'-phase			m'-phase			m''-phase		
	x	y	z	x	y	z	x	y	z
Hf_1	-0.6121	0.4581	0.5023	-0.0789	0.4445	0.5022	-0.0427	0.4036	0.4740
Hf_2	-0.0048	0.0086	0.0023	-0.0053	0.0035	0.0023	-0.0426	0.0121	-0.0259
Ce_1	0.4405	-0.0097	0.4939	0.5080	-0.0101	0.4377	0.4575	-0.0614	0.4739
Ce_2	0.4935	0.4765	-0.0061	0.4077	0.4582	-0.0623	0.4572	0.4772	-0.0259
O_1	0.6105	0.6362	0.6225	0.5569	0.6148	0.5902	0.7016	0.6443	0.6433
O_2	0.3235	0.8306	0.1225	0.3588	0.8332	0.0901	0.2130	0.6443	0.3048
O_3	0.0982	0.7727	0.6655	0.0367	0.6770	0.7750	0.2130	0.7715	0.8048
O_4	0.8358	0.6940	0.1655	0.8791	0.7710	0.2750	0.7015	0.7715	0.1432
O_5	0.2161	0.25888	0.7493	0.2058	0.1930	0.7117	0.1947	0.2563	0.7183
O_6	0.7179	0.2079	0.2493	0.7099	0.2550	0.2171	0.7200	0.2563	0.2297
O_7	0.7502	0.1913	0.7522	0.7430	0.1718	0.7271	0.7200	0.1594	0.7297
O_8	0.1838	0.2755	0.2522	0.1727	0.2762	0.2271	0.1946	0.1596	0.2183

Figure S5 | Structures of HCO superlattice. Visualization of atomic structures optimized o'-phase (A), m'-phase (B) and m''-phase (C) phases of superlattice. (D) atomic positions in the crystal coordinates of these structures.

Table S2 | Amplitudes of phonon modes which condensed in the different phases of HCO superlattice and bulk-HfO_2. The original modes condensed in the transition from cubic (c-phase) to low symmetry (t-, o- and m-phases) phases in HfO_2 are shown in black text, while additional modes which induce in their respective optimized phases in superlattice are shown in red text. The second row shows the atomic displacements of hafnium (Hf) and two unidentical oxygen (O_1 and O_2) atoms in their respective phonon modes (in same column in first row) in the primitive unit cell of cubic phase of HfO_2. Phonon modes which reverse their sign in down-polarized orthodromic D_1 and D_2 phases in the superlattice relative to up-polarized U phase are highlighted with blue background.

Phase		Γ_{15}^{z}	$X_2^{\prime x}$	X_5^{y}	Y_5^{z}	Z_5^{x}	$Z_5^{\prime y}$	X_1^{x}	Γ_{25}^{z}	Z_5^{y}	$X_5^{\prime y}$	$Y_5^{\prime z}$	$Z_5^{\prime x}$	$Y_3^{\prime y}$	$Z_5^{\prime x}$	$X_5^{\prime z}$	$Y_5^{\prime x}$	$Z_5^{\prime y}$
	Hf	0	0	0	0	0	0	0	0	0	0	0	0	u_y	u_x	u_z	u_x	u_y
	O_1	$-u_z$	$-u_x$	u_y	u_z	u_x	$-u_y$	u_x	u_z	u_y	$-u_y$	$-u_z$	$-u_x$	0	0	0	0	0
	O_2	$-u_z$	$-u_x$	$-u_y$	$-u_z$	$-u_x$	$-u_y$	$-u_x$	$-u_z$	$-u_y$	$-u_y$	$-u_z$	$-u_x$	0	0	0	0	0
c	Q(Å)	0	0	0	0	0	0	0	0	0	0	0	0	0	0	0	0	0
c'	Q(Å)	0	0	0	0	0	0	-0.115	0	0	0	0	0	0	0	0	0	0
t	Q(Å)	0	0.397	0	0	0	0	0	0	0	0	0	0	0	0	0	0	0
t'	Q(Å)	0	0.218	0	0	0	0	-0.119	0	0	0	0	0	0	0	0	0	0
o (U)	Q(Å)	0.385	0.503	0.380	0.385	0.358	0.110	0	0	0	0	0	0	0.083	0.174	0	0	0
o' (U)	Q(Å)	0.379	0.497	0.381	0.399	0.372	0.129	-0.089	0.086	0.139	0.077	0.075	0.042	0.085	0.149	0.022	0.005	0.048
o'(D_1)	Q(Å)	-0.379	0.497	-0.381	0.399	-0.372	0.129	-0.089	-0.086	0.139	-0.077	0.075	-0.042	-0.085	0.149	-0.022	0.005	-0.048
o' (D_2)	Q(Å)	-0.379	-0.497	0.381	-0.399	-0.372	0.129	-0.089	0.086	-0.139	-0.077	0.075	0.042	0.085	0.149	-0.022	0.005	0.048
m	Q(Å)	0	0.670	0	0	0.565	0	0	0.322	0.428	0.060	0.267	0	0.132	0	0.221	0.248	0
m'	Q(Å)	0.144	0.707	0.157	0.140	0.622	0.078	-0.090	0.380	0.451	0.138	0.322	0.043	0.120	0.031	0.173	0.255	0.036

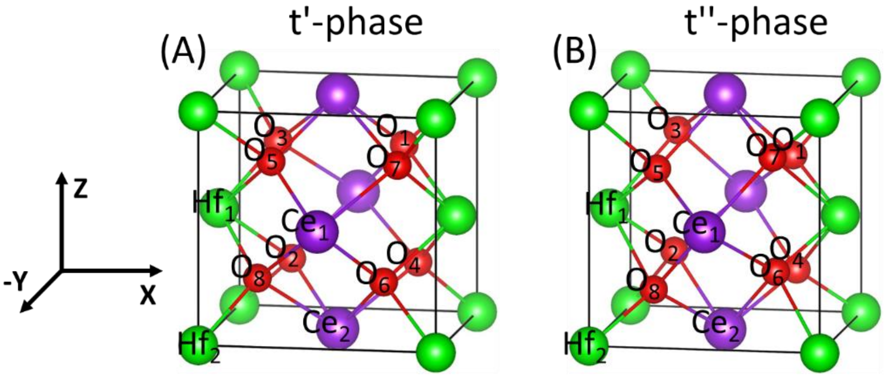

(C)

Atoms	t'-phase			t''-phase		
	x	y	z	x	y	z
Hf_1	0.0000	0.5000	0.5000	0.0000	0.5000	0.5000
Hf_2	0.0000	0.0000	0.0000	0.0000	0.0000	0.0000
Ce_1	0.5000	0.0000	0.5000	0.5000	0.0000	0.5000
Ce_2	0.5000	0.5000	0.0000	0.5000	0.5000	0.0000
O_1	0.7368	0.7500	0.7500	0.7576	0.7107	0.7114
O_2	0.2632	0.7500	0.2500	0.2424	0.7107	0.2886
O_3	0.2054	0.7500	0.7500	0.2424	0.7893	0.7886
O_4	0.7946	0.7500	0.2500	0.7576	0.7893	0.2114
O_5	0.2632	0.2500	0.7500	0.2424	0.2893	0.7114
O_6	0.7368	0.2500	0.2500	0.7576	0.2893	0.2886
O_7	0.7946	0.2500	0.7500	0.7576	0.2107	0.7886
O_8	0.2054	0.2500	0.2500	0.2424	0.2107	0.2114

Figure S6 | Tetragonal structures of HCO superlattice. Visualization of atomic structures optimized t'-phase (A) and t''-phase (B) phases of superlattice. (C) atomic positions in the crystal coordinates of these structures.

(A)

Phase		Γ_{25}^{y}	$Y_2^{\prime y}$	$Z_2^{\prime z}$	X_5^{z}	$Z_5^{\prime x}$	X_1^{x}	$Y_3^{\prime y}(Hf)$	$Z_5^{\prime y}(Hf)$
	Hf	0	0	0	0	0	0	u_y	u_y
	O_1	u_y	$-u_y$	u_z	u_z	u_x	u_x	0	0
	O_2	$-u_y$	$-u_y$	$-u_z$	$-u_z$	$-u_x$	$-u_x$	0	0
t"	Q(Å)	0	-0.299	-0.293	0	0	-0.056	0	0
m"	Q(Å)	0.060	-0.435	-0.329	0.285	-0.102	0.004	0.096	0.202

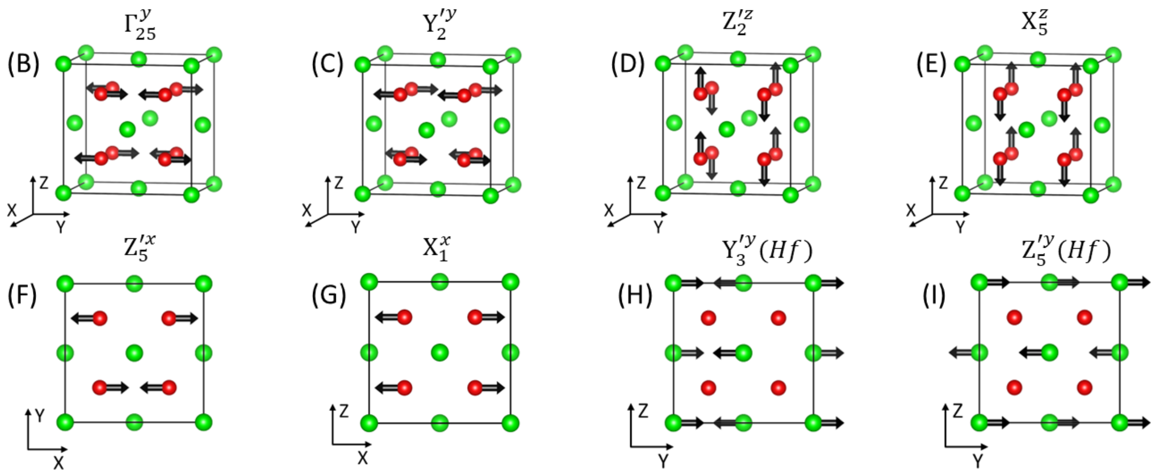

Figure S7 | Phonon modes condensed in t"- and m"-phases of HCO superlattice. (A) Amplitudes (in Å) of phonon modes which are condensed in the transformation of high-symmetry cubic phase into t"- and m"-phases. (B-I) Show the atomic displacements of these phonon modes in the high-symmetry cubic phases of HfO_2.

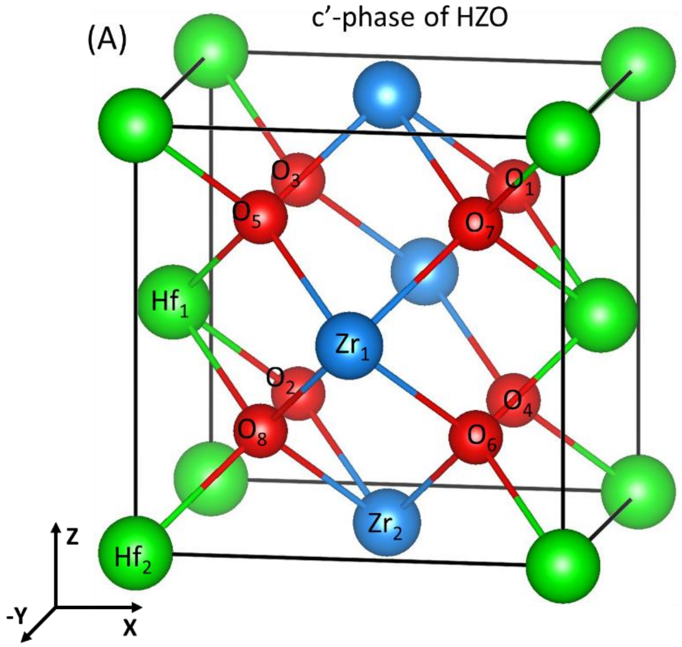

(B)

Atoms	c'-phase		
	x	y	z
Hf_1	0.0000	0.5000	0.5000
Hf_2	0.0000	0.0000	0.0000
Ce_1	0.5000	0.0000	0.5000
Ce_2	0.5000	0.5000	0.0000
O_1	0.7518	0.7500	0.7500
O_2	0.2482	0.7500	0.2500
O_3	0.2482	0.7500	0.7500
O_4	0.7518	0.7500	0.2500
O_5	0.2482	0.2500	0.7500
O_6	0.7518	0.2500	0.2500
O_7	0.7518	0.2500	0.7500
O_8	0.2284	0.2500	0.2500

Figure S8 | Cubic phase of HZO superlattice. (A) Visualization of atomic structure of c'-phase of HZO in its conventional unit cell of 12 atoms, and (B) its atomic positions in the crystal coordinates.

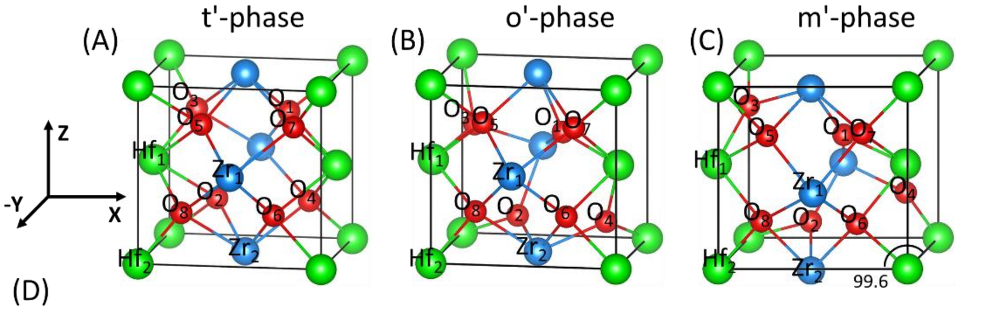

(D)

Atoms	t'-phase			o'-phase			m'-phase		
	x	y	z	x	y	z	x	y	z
Hf_1	0.0000	0.5000	0.5000	-0.0656	0.4659	0.4996	-0.0835	0.4480	0.5007
Hf_2	0.0000	0.0000	0.0000	-0.0004	0.0008	-0.0004	-0.0006	0.000	0..0007
Zr_1	0.5000	0.0000	0.5000	0.4354	-0.0011	0.5012	0.4989	0.0001	0.4148
Zr_2	0.5000	0.5000	0.0000	0.4986	0.4679	0.0012	0.4168	0.4480	-0.0851
O_1	0.6966	0.7500	0.7500	0.6018	0.6625	0.6405	0.5560	0.6544	0.6250
O_2	0.3036	0.7500	0.2500	0.3321	0.8042	0.1405	0.3597	0.7935	0.1250
O_3	0.1933	0.7500	0.7500	0.1007	0.7980	0.6452	0.05388	0.6588	0.7851
O_4	0.8066	0.7500	0.2500	0.8332	0.6687	0.1452	0.8619	0.7892	0.2851
O_5	0.3033	0.2500	0.7500	0.2354	0.2992	0.7495	0.2285	0.1740	0.7018
O_6	0.6966	0.2500	0.2500	0.6985	0.1975	0.2495	0.6873	0.2740	0.2018
O_7	0.8066	0.2500	0.7500	0.7395	0.1954	0.7498	0.7327	0.1734	0.7163
O_8	0.1933	0.2500	0.2500	0.1944	0.2713	0.2498	0.1830	0.2746	0.2163

Figure S9 | Low-symmetry phases of HZO. Visualization of atomic structures of optimized tetragonal (t'-phase) (A), orthorhombic (o'-phase) (B) and monoclinic (m'-phase) (C) phases of HZO. (D) atomic positions in the crystal coordinates of these structures.

Table S3 | Amplitudes of phonon modes which condensed in the different phases of HfO_2, ZrO_2 and HZO. The original modes condensed in the transition from cubic (c-phase) to low symmetry (t-, o- and m-phases) phases in HfO_2 are shown in black text, while weakly induced additional modes in their respective optimized phases in HZO are shown in red text. The second row shows the atomic displacements of hafnium (Hf) and two unidentical oxygen (O_1 and O_2) atoms in their respective phonon modes (in same column in first row) in the primitive cubic phase of HfO_2.

Phase		System	Γ_{15}^{z}	$X_2^{\prime x}$	X_5^{y}	Y_5^{z}	Z_5^{x}	$Z_5^{\prime y}$	X_1^{x}	Γ_{25}^{z}	Z_5^{y}	$X_5^{\prime y}$	$Y_5^{\prime z}$	$Z_5^{\prime x}$	$Y_3^{\prime y}$	$Z_5^{\prime x}$	$X_5^{\prime z}$	$Y_5^{\prime x}$	$Z_5^{\prime y}$
	Hf		0	0	0	0	0	0	0	0	0	0	0	0	u_y	u_x	u_z	u_x	u_y
	O_1		$-u_z$	$-u_x$	u_y	u_z	u_x	$-u_y$	u_x	u_z	u_y	$-u_y$	$-u_z$	$-u_x$	0	0	0	0	0
	O_2		$-u_z$	$-u_x$	$-u_y$	$-u_z$	$-u_x$	$-u_y$	$-u_x$	$-u_z$	$-u_y$	$-u_y$	$-u_z$	$-u_x$	0	0	0	0	0
c'	Q(Å)	HZO	0	0	0	0	0	0	-0.013	0	0	0	0	0	0	0	0	0	0
t	Q(Å)	HfO_2	0	0.397	0	0	0	0	0	0	0	0	0	0	0	0	0	0	0
t	Q(Å)	ZrO_2	0	0.421	0	0	0	0	0	0	0	0	0	0	0	0	0	0	0
t'	Q(Å)	HZO	0	0.409	0	0	0	0	-0.012	0	0	0	0	0	0	0	0	0	0
o	Q(Å)	HfO_2	0.385	0.503	0.380	0.385	0.358	0.110	0	0	0	0	0	0	0.083	0.174	0	0	0
o	Q(Å)	ZrO_2	0.391	0.520	0.365	0.378	0.345	0.111	0	0	0	0	0	0	0.081	0.163	0	0	0
o'	Q(Å)	HZO	0.391	0.510	0.375	0.386	0.357	0.111	-0.010	0.009	0.015	0.007	0.008	0.005	0.083	0.169	-0.004	0.003	0.005
m	Q(Å)	HfO_2	0	0.670	0	0	0.565	0	0	0.322	0.428	0.060	0.267	0	0.132	0	0.221	0.248	0
m	Q(Å)	ZrO_2	0	0.681	0	0	0.564	0	0	0.324	0.421	0.062	0.270	0	0.132	0	0.228	0.238	0
m'	Q(Å)	HZO	0.005	0.675	0.008	0.015	0.565	0.007	-0.010	0.322	0.424	0.061	0.269	0.005	0.132	0.001	0.224	0.243	0.000

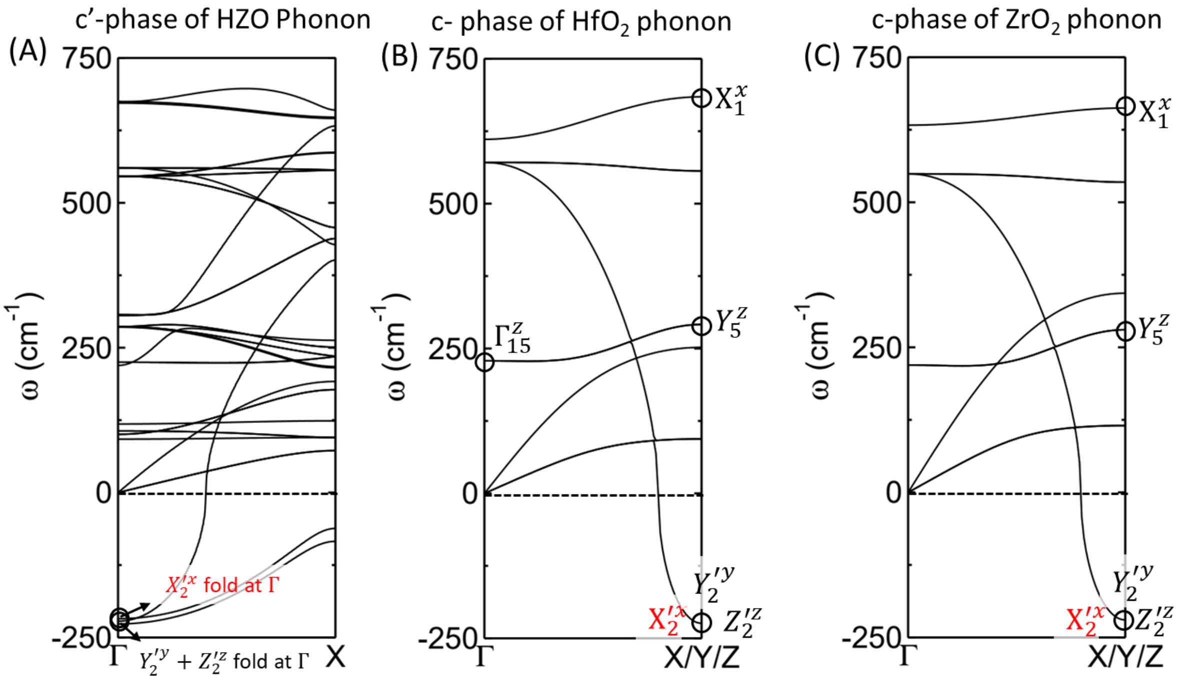

Figure S10 | Phonon spectra of cubic structures of HZO superlattice, HfO_2 and ZrO_2. (A) Phonon spectra of optimized cubic phases of HZO superlattice (conventional unit cell with 12 atoms: 4 hafnium and 8 oxygen), (B) HfO_2 (primitive unit cell with 3 atoms: 1 hafnium and 2 oxygen) and (C) ZrO_2 (primitive unit cell) and. The imaginary frequencies ($\omega^2 < 0$) depicted with negative sign in phonon spectra of these phases, which make them dynamically unstable at low temperature in their respective cubic phases. The instability of $X_2'^x$ mode in HZO does not reduces relative to HfO_2 because the same mode is also most unstable in the c-phase of ZrO2. Moreover, the $Y_2'^y + Z_2'^z$ mode in c'-phase of HZO also remain almost equally unstable relative to its $X_2'^x$ mode. Phonon modes which are involved in the cubic to orthorhombic phase transformation are shown in the phonon spectrum of HfO_2 and ZrO_2.

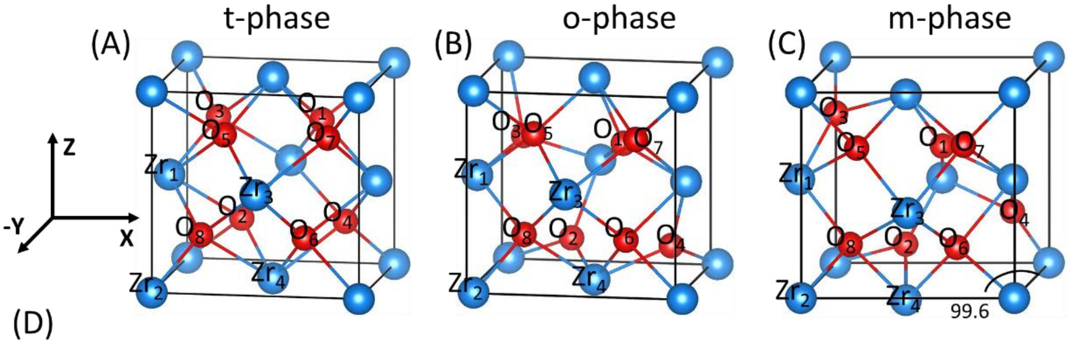

(D)

Atoms	t-phase			o-phase			m-phase		
	x	y	z	x	Y	z	x	y	z
Zr_1	0.0000	0.5000	0.5000	-0.0307	0.4840	0.5005	-0.0823	0.4480	0.5008
Zr_2	0.0000	0.0000	0.0000	0.0307	0.0159	0.0005	-0.0019	0.0001	0.0008
Zr_3	0.5000	0.0000	0.5000	0.4693	0.0159	0.5005	0.4981	0.0001	0.4138
Zr_4	0.5000	0.5000	0.0000	0.5307	0.4840	0.0005	0.4176	0.4480	-0.0862
O_1	0.6935	0.7500	0.7500	0.6350	0.6840	0.6446	0.5547	0.6572	0.6273
O_2	0.3064	0.7500	0.2500	0.3650	0.8159	0.1446	0.3610	0.7909	0.1273
O_3	0.1935	0.7500	0.7500	0.1350	0.8159	0.6446	0.0547	0.6572	0.7873
O_4	0.8064	0.7500	0.2500	0.8650	0.6840	0.1446	0.8610	0.7909	0.2873
O_5	0.3064	0.2500	0.7500	0.2733	0.2852	0.7488	0.2313	0.1745	0.7001
O_6	0.6935	0.2500	0.2500	0.7267	0.2147	0.2488	0.6845	0.2735	0.2001
O_7	0.8064	0.2500	0.7500	0.7733	0.2147	0.7588	0.7313	0.1745	0.7145
O_8	0.1935	0.2500	0.2500	0.2267	0.2852	0.2488	0.1845	0.2735	0.2145

Figure S11 | Low-symmetry phases of ZrO_2. Visualization of atomic structures of tetragonal (t-phase) (A), orthorhombic (B) and monoclinic (C) phases of ZrO_2. (D) atomic positions in the crystal coordinates of these structures.

Table S4. Structural parameters and energies of HfO_2, ZrO_2 and HZO superlattice estimated by first-principles calculations. Structural parameters and total energies of the cubic, tetragonal, orthorhombic, and monoclinic structures of HfO_2, ZrO_2 and their vertical superlattice HZO relative to their respective cubic structures and their polarizations.

Phase	System	a (Å)	b (Å)	c (Å)	γ^o	$E\text{-}E_{cubic}$ $(eV/u.c.)$	Polarization $(\mu C/cm^2)$
c	HfO_2	5.078	5.078	5.078	90	0	0
c	ZrO_2	5.118	5.118	5.118	90	0	0
c'	ZHO	5.0982	5.0980	5.0980	90	0	0
t	HfO_2	5.227	5.082	5.082	90	-0.39	0
t	ZrO_2	5.279	5.123	5.123	90	-0.41	0
t'	ZHO	5.254	5.101	5.101	90	-0.40	0
o	HfO_2	5.273	5.055	5.085	90	-0.72	56
o	ZrO_2	5.323	5.101	5.128	90	-0.56	57
o'	ZHO	5.297	5.076	5.106	90.02	-0.64	57
m	HfO_2	5.329	5.147	5.199	99.68	-1.04	0
m	ZrO_2	5.371	5.189	5.250	99.66	-0.85	0
m'	ZHO	5.350	5.167	5.223	99.65	-0.95	1

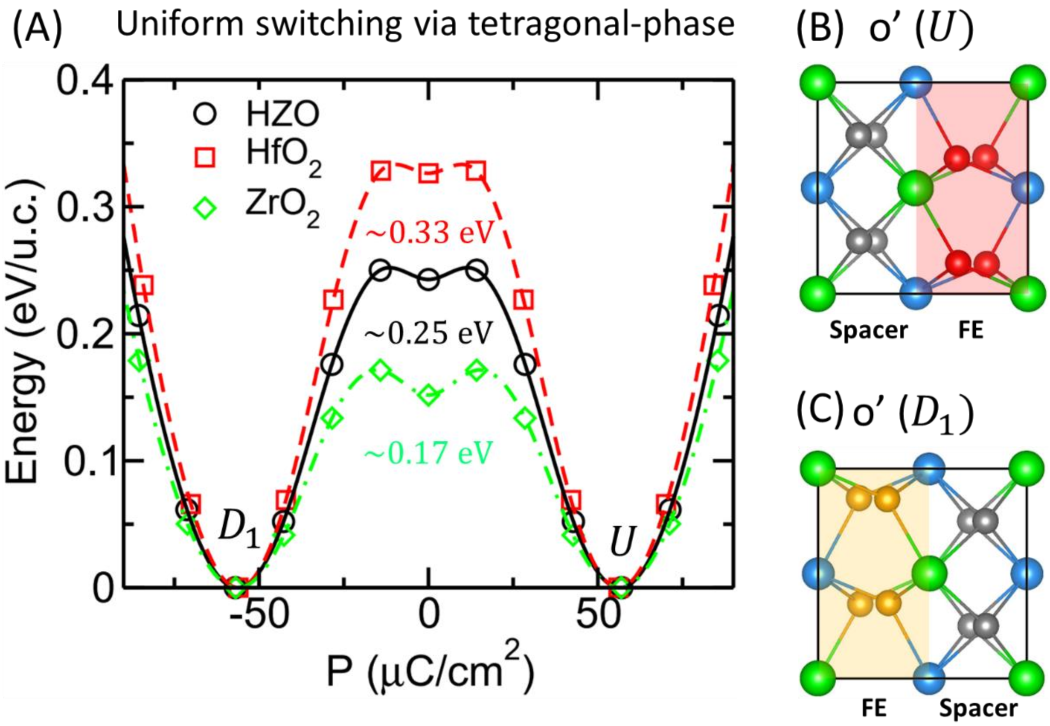

Figure S12 | Uniform polarization switching in HZO, HfO_2 and ZrO_2 via intermediate tetragonal phase. (A) The energy barriers during uniform polarization switching between up-polarized U (B) and down-polarized D_1 (C) of HZO (solid black line), HfO_2 (dashed red line) and ZrO_2 (dashed-dot green line) via their respective intermediate tetragonal phases. The energy barrier of the HZO (0.25 eV/u.c.) is an average of bulk HfO_2 (0.33 eV/u.c.) and ZrO_2 (0.17eV/u.c). The ferroelectric (FE) layers in crystal structures of U and D_1 are shown by red and yellow oxygen, respectively. While spacer layers are shown by silver oxygen atoms.

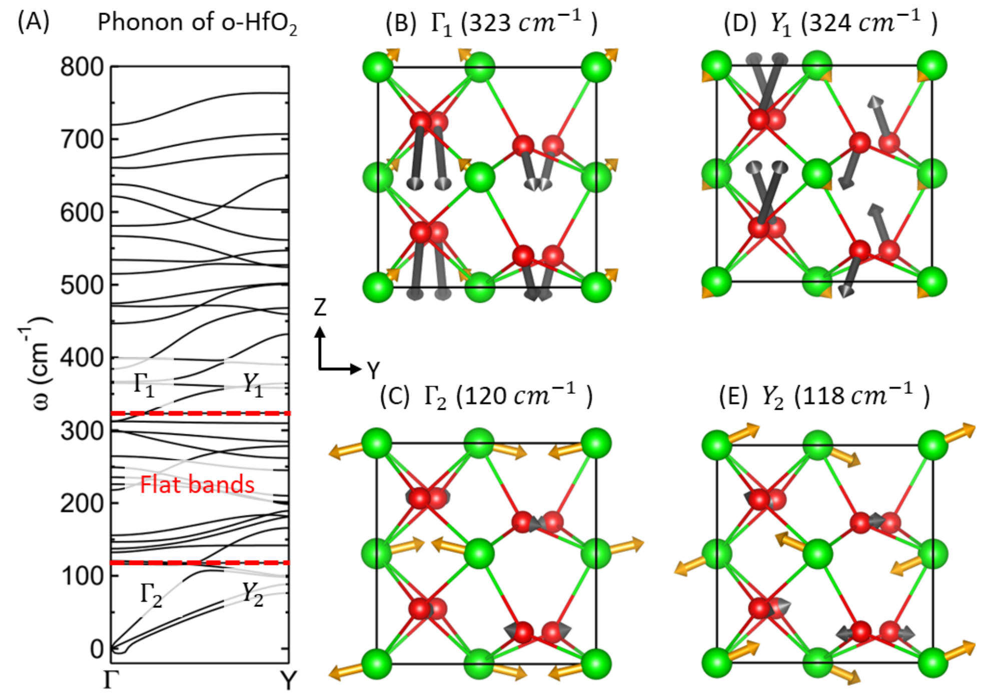

Figure S13 | Flat-phonon bands in polar phase of bulk-HfO_2. (A) The phonon spectrum of orthorhombic phase of bulk HfO_2 shows two flat phonon bands which involve the atomic displacements of all the modes condensed during cubic to orthorhombic phase transition. (B-C) The atomic displacements of flat band phonon at Γ point, and (D-E) at Y-point in the o-phase of HfO_2.

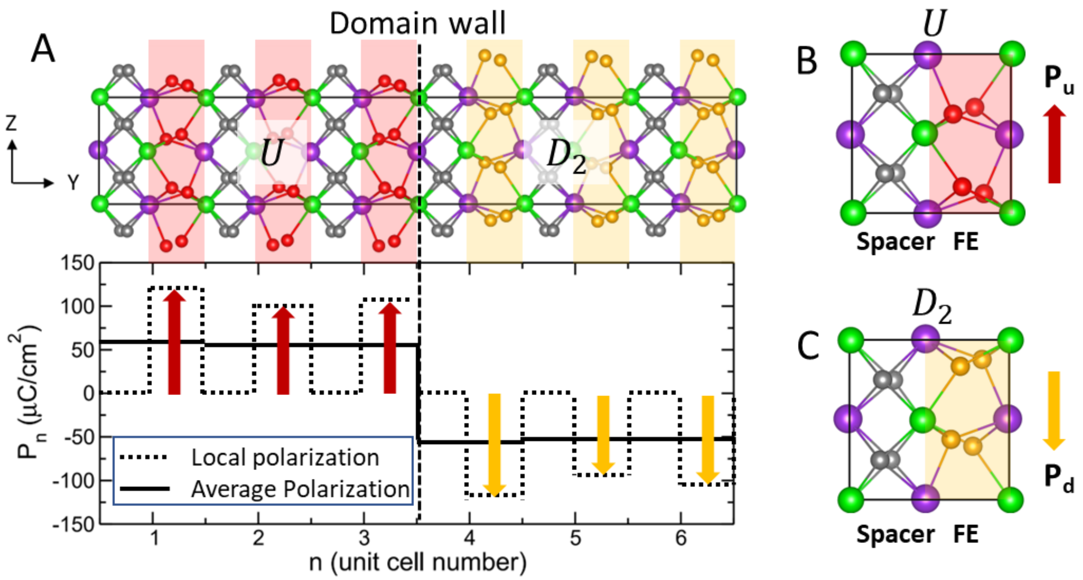

Figure S14 | Results of zero width domain wall in superlattice. (A) The atomic structure of 1x6x1 supercell of U/D_2 domain walls (top) along the y-direction. The silver oxygen atoms are in spacer layer, while red and yellow are in up- and down-polarized layers, respectively, while Hf and Ce atoms are shown in green and purple color, respectively. The local (dotted black line) and average (solid black line) polarization (bottom figure A) do not suppress across the domain wall and remain similar to the bulk domain. (B-C) Atomic structures of orthorhombic U and D2 phases in their respective 12 atoms single unit cells.

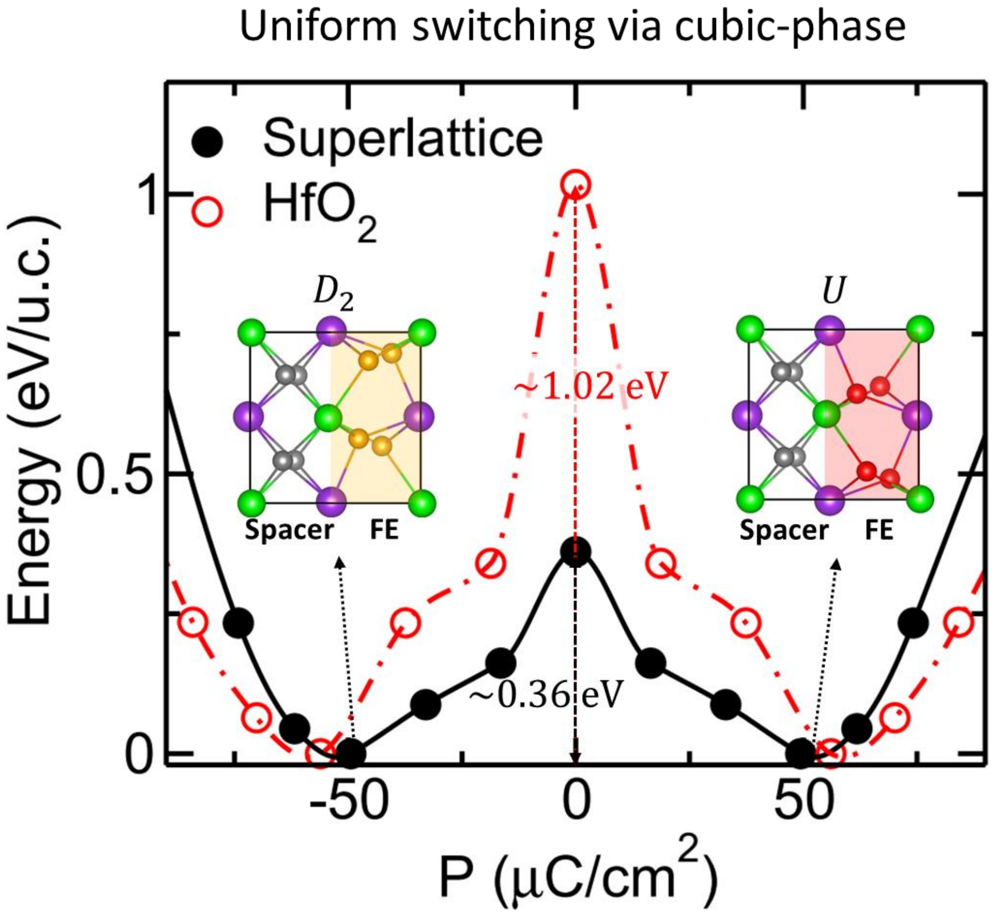

Figure S15 | Uniform polarization switching via intermediate cubic-like. The comparison of energy barrier during uniform polarization switching between down-polarized D_2 (left inset) and up-polarized U (right inset) of superlattice (solid black line) and their counterparts in bulk-HfO_2 (dash-dot red line) via intermediate cubic-like phase. The energy barrier in the superlattice reduces to 0.36 eV/u.c. from 1.02 eV/u.c. in the bulk HfO_2. However, this barrier is quite large relative to the energy difference between their respective cubic and orthorhombic phases. This is because the lattice parameters do not relax in the NEB calculations. Thus, in cubic-like phase the lattice parameters are of orthorhombic phase, while the atoms occupy their high-symmetry positions of their respective cubic phase.

References

1. Reyes-Lillo, S. E., Garrity, K. F. & Rabe, K. M. Antiferroelectricity in thin-film ZrO2 from first principles. *Phys Rev B Condens Matter Mater Phys* **90**, (2014).
